# The optimal timing for definitive operative stabilization of pelvic fractures in polytrauma patients: effects on clinical outcomes – a systematic review

**DOI:** 10.1007/s00068-025-02774-1

**Published:** 2025-02-07

**Authors:** Julia Dormann, Klemens Horst, Karolina Dahms, Eva Steinfeld, Kelly Ansems, Heidrun Janka, Maria-Inti Metzendorf, Thomas Breuer, Carina Benstoem, Frank Hildebrand, Eftychios Bolierakis

**Affiliations:** 1 Department of Intensive Care Medicine and Intermediate Care, Medical Faculty, RWTH Aachen University, Pauwelsstr. 30, D-52074 Aachen, Germany; 2 Department of Orthopaedics, Trauma and Reconstructive Surgery, Medical Faculty, RWTH Aachen University, Aachen, Germany; 3 Institute of General Practice, Medical Faculty of the Heinrich-Heine-University Dusseldorf, Dusseldorf, Germany

**Keywords:** Polytrauma, Intensive care unit, Early stabilization, Late stabilization, Pelvic fracture

## Abstract

**Purpose:**

The optimal timing for definitive surgical treatment of pelvic fractures in polytrauma patients remains a topic of ongoing discussion due to the complexity of these injuries. This analysis therefore aims to systematically compare early versus late definitive operative stabilization of pelvic fractures on outcome in polytrauma patients.

**Methods:**

PubMed, CENTRAL and Web of Science were systematically searched to identify relevant completed and ongoing studies from the inception of each database to March 13, 2023. Systematic reviews, randomized control trials (RCTs) and observational studies comparing early (< 24 h) versus late (> 24 h) definitive operative stabilization in adult polytrauma patients admitted to the ICU were included.

**Results:**

Since no systematic reviews and RCTs were available on this subject, one observational study was identified, including a total of 418 polytrauma patients (n_early_ = 165, n_late_ = 253), median age: 40.3 years (early 40.1 years, late 40.4 years). Early definitive stabilization was associated with a decreased risk of acute respiratory distress syndrome (ARDS) compared to late stabilization of unstable pelvis and acetabulum fractures (RR 0.38, 95% CI 0.18–0.81; RD 78 fewer per 1000, 95% CI 104 fewer to 24 fewer; 1 study, 418 participants; very low certainty of evidence). Furthermore, early definitive stabilization may decrease the risk of pneumonia compared to late stabilization of unstable pelvis and acetabulum fractures (RR 0.50, 95% CI 0.28–0.88; RD 85 fewer per 1000, 95% CI 122 fewer to 20 fewer); 1 study, 418 participants; very low certainty of evidence).

**Conclusion:**

There is limited evidence regarding early definitive fracture repair (≤ 24 h) compared to late repair of pelvic fractures in polytrauma patients. One observational study showed a reduced incidence of septic respiratory complications, ARDS, and multi-organ failure (MOF) in polytrauma patients who received early definitive fracture repair.

**Supplementary Information:**

The online version contains supplementary material available at 10.1007/s00068-025-02774-1.

## Introduction

The incidence of pelvic injuries in polytrauma patients ranges up to 25% [1]. Traumatic pelvic injuries, responsible for life threatening hemodynamic instability [2–4], are associated with high mortality and morbidity rates in polytraumatized patients. Therefore, applying the most beneficial treatment strategies in a timely fashion is of utmost importance [5, 6].

Typically, major pelvic injuries are stabilized via the Damage Control Orthopaedics (DCO) concept, where temporary external fracture fixation and patient resuscitation are the priority. Definitive fracture repair follows the physical stabilization of the patient [7] which includes restoration of stable hemodynamics, preserving normothermia, guaranteeing balanced hemostasis, and monitoring physiologic lactate or base-excess levels [4, 8–10]. Although this concept has been widely implemented in clinical settings, the optimal timing for conversion from temporary to definitive surgical fracture treatment continues to be controversial [11].

Due to the limited availability of robust evidence, comprehensive trials, or clear guidelines that focus on the optimal timing for definitive surgical fracture management of pelvic injuries in adult polytrauma patients, this systematic literature review aims to investigate the effects of the timepoint for definitive fracture fixation of pelvic injuries on clinical outcomes in polytrauma patients.

## Methods

This review is part of the guideline project ‘S3-Leitlinie Intensivmedizin nach Polytrauma’ (AWMF Nr. 040 − 014) guided by the German Interdisciplinary Association of Critical Care and Emergency Medicine (Deutsche Interdisziplinäre Vereinigung für Intensiv- und Notfallmedizin, DIVI) and the German Society for Anaesthesiology and Intensive Care Medicine (Deutsche Gesellschaft für Anästhesiologie und Intensivmedizin, DGAI). The aim was to summarize the current evidence in the field of polytrauma to formulate specific recommendations. All studies that were carried out as part of this project used the same methodology which was consented within the guideline group.

### Eligibility criteria

We included studies that compared definitive surgical stabilization versus late definitive surgical stabilization of pelvic injuries in adult polytrauma patients admitted to the intensive care unit (ICU) is, that met the following inclusion criteria:

- age of the included patients is ≥ 18 years.
- polytrauma is present and is defined as: a simultaneous injury to multiple body regions or organ systems, at least one or more of which, in combination, is life-threatening [12].
- the study type is a randomized controlled trial (RCT) or systematic review that includes RCTs or prospective cohort studies with *n* ≥ 300 (*n* ≥ 150 participants per intervention arm) if no RCTs could be found.
- the language of publication is English or German.
- it is not a multiple publication without additional information.
- the publication can be obtained as a full text.
- definitive surgical stabilization (< 24 h) versus late definitive surgical stabilization (> 24 h) of pelvic injuries in adult polytrauma patients admitted to the ICU.

### Search strategy

A systematic search developed by two experienced information specialists [MIM, HJ] was conducted in the following sources from inception of each database to March 13, 2023:

- MEDLINE (PubMed).
- Cochrane Central Register of Controlled Trials.
- Web of Science (Science Citation Index Expanded und Emerging Sources Citation Index).

Details of the implemented search strategy are provided in the Appendix No 1. In addition, reference lists of included studies were screened to identify other potentially eligible studies.

### Selection process

We imported citations from the systematic search into Rayyan [13]. Three authors independently screened the titles and abstracts of all potential studies [EB, KH and JD]. Full-text study publications were retrieved, imported into Excel and screened by two authors [EB and KH] independently. Reasons for exclusion of ineligible studies were recorded. Any disagreements were resolved through discussion or, if required, consultation with a third author [FH].

### Data collection process

A customised data collection form (Microsoft Excel) was used to collect study data [14]. The following data were extracted:

1. Study characteristics: authors, publication date, and study design.
2. Participants characteristics: number of included participants, gender, age.
3. Intervention: early definitive operative stabilization (< 24 h), late definitive operative stabilization (> 24 h).
4. Outcomes: all-cause mortality (day 28, day 60, time to-event, and up to longest follow-up), clinical status, serious adverse events (SAE), adverse events (AE), multiple organ failure (MOF), acute respiratory distress syndrome (ARDS), pneumonia, complications, length of stay, quality of life.

Extraction of study characteristics and outcome data of included studies was conducted by one author and checked by another [JD, KD]. Any disagreements were resolved by discussion or by consulting a third review author if necessary [KA]. Two authors [JD, KD] transmitted the outcome data into the Cochrane statistical software RevMan 5.3 which was checked by a third author [KA] [15]. Missing data resulted in the exclusion of the study in the analyses of the missing outcome.

### Study risk of bias assessment

Two authors [JD, KD] independently assessed the risk of bias of included studies using the ROBINS I tool [16]. ROBINS I addresses seven domains, including confounding and selection of participants for the study, classification of interventions, departures from intended interventions, missing data, measurement of outcomes, and selection of the reported result. The signaling questions recommended in the tool were used to make a judgment according to the available options. Algorithms proposed in ROBINS I were used to assign each domain and the overall risk of bias, a level of bias (low risk of bias, moderate risk of bias, serious risk of bias, critical risk of bias). We resolved any disagreements by discussion or by involvement of another author [KA].

### Synthesis methods

To summarize demographics, we used descriptive statistics. A meta-analysis was performed only, if the clinical and methodological characteristics of individual studies were sufficiently homogeneous. For all analyses, we used Rev-Man 5.3 [15]. Data entry into the Rev-Man software was checked by a second review author for accuracy. Outcome data were pooled using the random-effects model, as we anticipated that true effects would be related, but not the same for the studies included in our review. For dichotomous data, we performed analyses using the Mantel–Haenszel method under a random-effects model to report pooled risk ratios (RR) with 95% confidence intervals (CI). For continuous outcomes, we calculated mean differences with 95% CIs. Forest plots were provided to summarize the effects from individual studies. When data was lacking or incomplete for analysis, the corresponding information was reported narratively. A *p*-value of > 0.05 was considered as statistically significant. The data underwent analysis utilizing the Cochrane methodology.

### Certainty assessment

We used GRADE pro to create a summary of findings table and evaluated the certainty of the evidence using the GRADE approach [17].

## Results

### Study selection

**Fig. 1:**
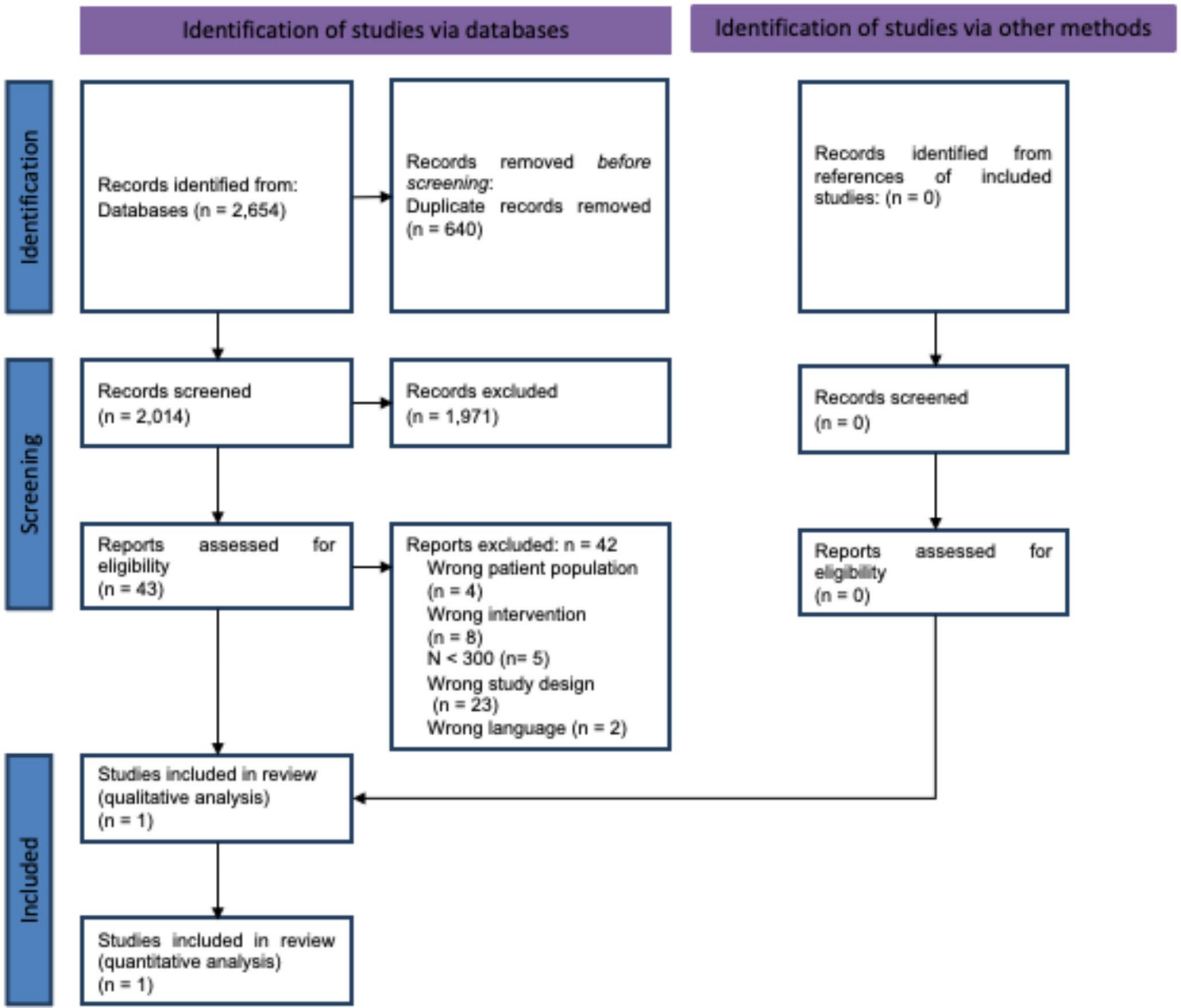
Flowchart of the systematic review selection process

The search identified 2,654 records. After removing duplicates, we screened 2,014 records based on title and abstract. We screened the full texts of the remaining 43 references. No additional full texts were identified. 42 records were excluded for different reasons (Fig. 1). One observational study was included in our analysis: Vallier et al. (2010) [18].

### Study characteristics

The study included [18] reported on 645 adult participants (mean age 40.3 years, 74% male). The total number of participants with polytrauma (ISS > 18) was 418. The included study used a retrospective design. Additional study characteristics are provided in Table 1.

**Table 1:** Study characteristics

Study (year)	Study design	No. of patients	Mean age (range) in years	Gender (m/f)	Intervention	Control
Vallier et al. (2010)	Retrospective review	*N* = 645 Early fixation Total: *N* = 233 ISS > 18: *N* = 165 Late fixation Total: *N* = 412 ISS > 18: *N* = 253	40.3 (16–86) Early fixation 40.1 (16–86) Late fixation 40.4 (16–82)	478/167 Early fixation 181/52 Late fixation 297/115	Early fixation within 24 h of pelvic ring fracture and/or acetabulum fractures	Late fixation of pelvic ring fracture and/or acetabulum fractures

### Risk of bias assessment

The overall risk of bias was critical due to bias in selection of participants into the study, bias in selection of the reported result and moderate bias due to confounding.

### Results of individual studies

The outcomes of early definitive stabilization were compared to late stabilization of unstable pelvis and acetabulum fractures in adult polytrauma patients admitted to the ICU (Table 2).

**Table 2:** Summary of findings table

Outcomes	№ of participants (studies) Follow-up	Certainty of the evidence (GRADE)	Relative effect (95% CI)	Anticipated absolute effects	
				Risk with late fixation	Risk difference with early fixation
Multiple organ failure	418 (1 observational study)	⨁◯◯◯ Very low^a, c^	**RR 0.42** (0.12 to 1.48)	43 per 1.000	**25 fewer per 1.000** (38 fewer to 21 more)
ARDS	418 (1 observational study)	⨁◯◯◯ Very low^a, b^	**RR 0.38** (0.18 to 0.81)	126 per 1.000	**78 fewer per 1.000** (104 fewer to 24 fewer)
Pneumonia	418 (1 observational study)	⨁◯◯◯ Very low^a, b^	**RR 0.50** (0.28 to 0.88)	170 per 1.000	**85 fewer per 1.000** (122 fewer to 20 fewer)
Pulmonary complication	418 (1 observational study)	⨁◯◯◯ Very low^a, b^	**RR 0.50** (0.32 to 0.79)	253 per 1.000	**126 fewer per 1.000** (172 fewer to 53 fewer)
Any complications	418 (1 observational study)	⨁◯◯◯ Very low^a, b^	**RR 0.58** (0.38 to 0.88)	261 per 1.000	**110 fewer per 1.000** (162 fewer to 31 fewer)

CI: confidence interval; RR: risk ratio

a. Critical risk of bias due to confounding bias; inadequate selection of participants and selection of the reported results

b. Imprecision due to only one study

c. Imprecision due to wide CI, only one study

### Multiple organ failure

**Fig. 2:**
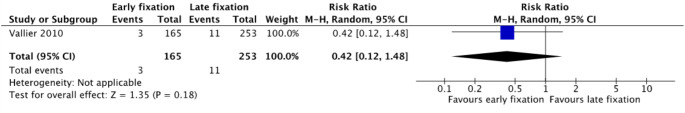
Forest plot describing the difference between early fixation compared to late fixation of unstable pelvis and acetabulum fractures regarding MOF

One study reported MOF for 418 participants (Fig. 2). We found that early definitive stabilization may makes little or no difference regarding MOF compared to late stabilization of unstable pelvis and acetabulum fractures (RR 0.42, 95%, CI 0.12–1.48; risk difference (RD) 22 fewer per 1000, 95% CI 38 fewer to 21 more; 1 study, 418 participants; very low certainty of evidence). The reason for downgrading was imprecision due to wide CI’s and only one study and a critical risk of bias.

### Acute respiratory distress syndrome

**Fig. 3:**
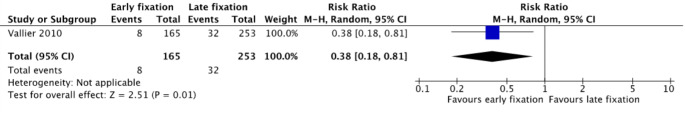
Forest plot describing the difference between early fixation compared to late fixation of unstable pelvis and acetabulum fractures regarding ARDS

One study reported ARDS for 418 participants (Fig. 3). We found that early definitive stabilization probably decreases the risk of ARDS compared to late stabilization of unstable pelvis and acetabulum fractures (RR 0.38, 95% CI 0.18–0.81; RD 78 fewer per 1000, 95% CI 104 fewer to 24 fewer; 1 study, 418 participants; very low certainty of evidence). The reason for downgrading was imprecision due to only one study and a critical risk of bias.

### Pneumonia

**Fig. 4:**
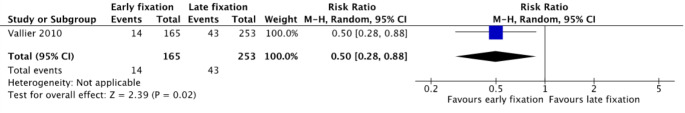
Forest plot describing the difference between early fixation compared to late fixation of unstable pelvis and acetabulum fractures regarding pneumonia

One study reported pneumonia for 418 participants (Fig. 4). We found that early definitive stabilization probably decreases the risk of pneumonia compared to late stabilization of unstable pelvis and acetabulum fractures (RR 0.50, 95% CI 0.28–0.88; RD 85 fewer per 1000, 95% CI 122 fewer to 20 fewer; 1 study, 418 participants; very low certainty of evidence). The reason for downgrading was imprecision due to only one study and a critical risk of bias.

### Complications

**Fig. 5:**
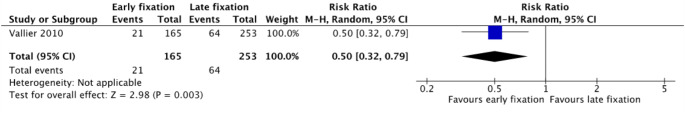
Forest plot describing the difference between early fixation compared to late fixation of unstable pelvis and acetabulum fractures regarding pulmonary complications

One study reported pulmonary complications (ARDS, pneumonia and pulmonary embolism) for 418 participants (Fig. 5). We found that early definitive stabilization probably decreases the risk of pulmonary complications compared to late stabilization of unstable pelvis and acetabulum fractures (RR 0.50, 95% CI 0.32–0.79; RD 126 fewer per 1000, 95% CI 172 fewer to 53 fewer; 1 study, 418 participants; very low certainty of evidence). The reason for downgrading was imprecision due to only one study and a critical risk of bias.

**Fig. 6:**
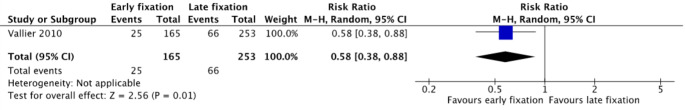
Forest plot describing the difference between early fixation compared to late fixation of unstable pelvis and acetabulum fractures regarding any complications

One study reported any complications (infections, pulmonary complications, deep venous thrombosis, organ failure and death) for 418 participants (Fig. 6). We found that early definitive stabilization probably decreases the risk of any complications compared to late stabilization of unstable pelvis and acetabulum fractures (RR 0.58, 95% CI 0.32–0.88; RD 110 fewer per 1000, 95% CI 162 fewer to 31 fewer; 1 study, 418 participants; very low certainty of evidence). The reason for downgrading was imprecision due to only one study and a critical risk of bias.

We found no data regarding mortality, clinical status, serious adverse events, adverse events, length of stay and quality of life.

## Discussion

Pelvic injuries are common in polytraumatized patients. As mortality and morbidity rates remain high, correct treatment strategies are of the highest importance. This analysis focused on early versus late definitive fixation of pelvic fractures. High quality literature regarding the optimal time point for definitive surgical stabilization is sparse. As a consequence, only one study reporting on 418 adult polytrauma patients was identified [18]. The main findings of this study can be summarized as follows:

A) Early definitive fracture repair conducted within 24 h resulted in a reduced incidence of septic respiratory complications, ARDS, and MOF.
B) Early intervention may improve the clinical outcome of pelvic injuries, highlighting the need for prompt definitive care.

While definitive evidence is lacking, the presented analysis indicates that early surgical treatment of pelvic injuries in polytrauma patients offers potential advantages. In line with the presented results, also Taylor et al. demonstrated in a retrospective cohort of less than 300 participants that early definitive pelvic fracture repair (within 72 h) in polytrauma patients leads to a reduced length of hospitalisation [19]. This has potential implications for healthcare resource management and may suggest a quicker recovery and improved quality of life for patients returning to their daily activities. Yet, the absence of a commonly agreed definition of “early definitive care” adds complexity to the interpretation of findings. The timeframe for definitive surgical care in studies showed considerable variation, ranging from 24 to 72 h [2, 18, 19–21, 22, 23]. The unstandardized definition creates difficulties in making direct comparisons between studies and obstructs the formulation of precise clinical guidelines. However, Devaney et al.‘s investigation also supports the idea of timely definitive treatment of fractures [20]. In their 10-year retrospective analysis of 1,270 patients with pelvic ring and acetabular fractures, the researchers found no clear disadvantage for early surgical intervention [20]. This means that patients who received earlier definitive osteosynthetic care did not experience increased mortality, extended periods of intensive care observation, or prolonged hospital stays. These results are significant in allaying apprehensions that hastening surgical management might unintentionally yield adverse consequences. However, the proportion of polytrauma patients was too low to be included in our analysis.

The benefits of early definitive surgical treatment of pelvic fractures revolve around the reduction or prevention of complications and the acceleration of patient rehabilitation. Patients suffering from multiple trauma, including pelvic injuries, are especially vulnerable to a range of adverse events such as pulmonary, septic, and thromboembolic complications. Early surgical intervention seems to reduce these risks, which is vital in a population already coping with multiple traumatic injuries. The potential decrease in septic pulmonary complications and ARDS, as proven by Vallier et al., is a crucial statement. These complications can significantly affect patient outcomes and are frequently demanding to handle.

Although current evidence favors early definitive surgical treatment, care must be taken when interpreting these findings. While the data is persuasive, it is mainly based on observational studies that are inherently biased and limited. These studies frequently lack the stringent controls and randomisation present in RCTs, making it difficult to establish causation and draw definitive conclusions.

Current evidence, although limited, suggests potential benefits associated with early definitive surgical treatment of pelvic fractures in polytrauma patients. These benefits include faster and safer remobilisation, reduced complications, and accelerated rehabilitation [2, 18, 19, 21, 24–26].

The presented analysis revealed a significant absence of high-quality evidence, such as RCTs, to inform decision-making regarding the optimal timing of definitive surgical intervention. This deficiency in knowledge represents a critical issue, as RCTs are generally considered the gold standard for establishing treatment guidelines. Without such rigorous studies, the foundation for informed decisions weakens. A cut-off at 300 patients for observational studies was established, as this threshold was determined during the development of the guideline from which this review originated. This decision was made by an experienced, interdisciplinary expert group, ensuring that only studies with sufficient sample sizes were considered to enhance the reliability of the evidence. However, this criterion also led to the exclusion of many smaller studies, potentially omitting relevant data and further highlighting the need for well-designed RCTs to fill this gap and provide robust data for clinical practice.

Given these limitations, additional research is necessary to provide more comprehensive and conclusive findings regarding the most appropriate time for definitive surgical intervention in polytrauma patients with pelvic injuries. To establish a more precise evidence base and enable the formulation of accurate recommendations, high-quality randomised controlled trials are required. Additionally, it is necessary to reach a consensus on the definitions and criteria for “early definitive care” to ensure consistency across future studies and achieve more substantial comparisons.

## Conclusion

There is limited evidence regarding early definitive fracture repair (≤ 24 h) compared to late repair in polytrauma patients with pelvic injuries. While one observational study suggests a reduced incidence of septic respiratory complications, ARDS, and MOF in polytrauma patients who received early definitive fracture repair, the overall evidence base remains inusfficient. This highlights the need for more high-quality well-structured controlled trials on this subject. Furthermore, a standardized definition of “early” and “late” fixation could allow appropriate pooling of the corresponding results in the context of meta-analysis.

## Electronic supplementary material

Below is the link to the electronic supplementary material.

Supplementary Material 1

## Data Availability

Additional study data are available from the corresponding author.

## Abbreviations

AE: Adverse event
ARDS: Acute respiratory distress syndrome
CI: Confidence intervals
DCO: Damage Control Orthopaedics
DGAI: German Society for Anaesthesiology and Intensive Care Medicine (Deutsche Gesellschaft für Anästhesiologie und Intensivmedizin)
DIVI: German Interdisciplinary Association of Critical Care and Emergency Medicine (Deutsche Interdisziplinäre Vereinigung für Intensiv- und Notfallmedizin)
ICU: Intensive care unit
MOF: Multiple organ failure
RCT: Randomized controlled trial
RD: Risk difference
RR: Risk ratio
SAE: Serious adverese event
