# Supplementary material for: The optimal timing for definitive operative stabilization of pelvic fractures in polytrauma patients: effects on clinical outcomes – a systematic review
Source: Eur J Trauma Emerg Surg. 2025 Feb 7;51(1):100. doi: 10.1007/s00068-025-02774-1 (PMC11805852; doi:10.1007/s00068-025-02774-1)
Supplement: Supplementary file 1 — Supplementary Material 1 [file 68_2025_2774_MOESM1_ESM.docx]

Appendix No. 1 Search Strategy

1. PubMed

#1

("Pelvic Bones"[mh] OR pelvic[tw] OR "hip bone*"[tw] OR "coxal bone*"[tw] OR "innominate bone*"[tw] OR ilium[tw] OR ischium[tw] OR "pubic bone*"[tw])

#2

"Fracture Fixation"[mh] OR "fixation*"[tw] OR osteosynthe*[tw] OR "stabilization"[tw] OR "stabilisation"[tw] OR surgic*[tiab] OR surger*[tiab] OR operati*[tiab]

#3

"Multiple Trauma"[mh] OR "Trauma Centers"[mh] OR polytrauma*[tw] OR "multiple trauma*"[tw] OR "major trauma*"[tw] OR "multiple injur*"[tw] OR "multisystem trauma"[tw] OR "multisystem injur*"[tw] OR "multitrauma"[tw] OR "trauma patient*"[tw] OR "trauma population"[tw] OR "trauma care"[tw] OR "trauma cent*"[tw] OR "Critical Illness"[mh] OR "Critical Care"[mh:noexp] OR "critical illness*"[tw] OR "critically ill*"[tw] OR "critical care"[tw] OR "intensive care"[tw] OR (blunt[tiab] AND trauma[tiab])

#4

[Cochrane Handbook RCT-Filter, sensitivity max. version]^1^

(randomized controlled trial[pt] OR controlled clinical trial[pt] OR randomized[tiab] OR placebo[tiab] OR drug therapy[sh] OR randomly[tiab] OR trial[tiab] OR groups[tiab]) NOT (animals[mh] NOT humans[mh])

#5

[Search filter for Non-randomized studies, sensitivity max. version]^2^

(cohort[all] OR (control[all] AND study[all]) OR (control[tw] AND group*[tw]) OR epidemiologic studies[mh] OR program[tw] OR clinical trial[pt] OR comparative stud*[all] OR evaluation studies[all] OR statistics as topic[mh] OR survey*[tw] OR follow-up*[all] OR time factors[all] OR ci[tw]) NOT ((animals[mh:noexp] NOT humans[mh:noexp]) OR comment[pt] OR editorial[pt] OR review[pt] OR meta analysis[pt] OR case report[tw] OR consensus[mh] OR guideline[pt] OR history[sh])

#6 [pelvic + fracture + polytrauma + RCT]

#1 AND #2 AND #3 AND #4

= 373 RCTs

#7 [pelvic + fracture + polytrauma + Observational Studies, sensitivity max]

#1 AND #2 AND #3 AND #5

= 1402 Observational Studies

2. CENTRAL (via Cochrane Register of Studies Online)

#1

(pelvic OR hip bone* OR coxal bone* OR innominate bone* OR ilium OR ischium OR pubic bone*):TI,AB,KY

#2

(fixation* OR fracture care OR osteosynthe* OR stabilization OR stabilisation OR surgic* OR surger* OR operati*):TI,AB,KY

#3

(polytrauma* OR multiple trauma* OR major trauma* OR multiple injur* OR multisystem trauma OR multisystem injur* OR multitrauma OR trauma patient* OR trauma population OR trauma care OR trauma cent* OR critical illness* OR critically ill* OR critical care OR intensive care OR (blunt AND trauma)):TI,AB,KY

#1 AND #2 AND #3

= 64 RCTs

3. Web of Science (Science Citation Index Expanded und Emerging Sources Citation Index)

#1

TI=(pelvic OR "hip bone*" OR "coxal bone*" OR "innominate bone*" OR ilium OR ischium OR "pubic bone*") OR AB=(pelvic OR "hip bone*" OR "coxal bone*" OR "innominate bone*" OR ilium OR ischium OR "pubic bone*")

#2

TI=(fixation OR "fracture care" OR osteosynthe* OR stabilization OR stabilisation OR surgic* OR surger* OR operati*)) OR AB=(fixation OR "fracture care" OR osteosynthe* OR stabilization

#3

TI=((polytrauma* OR "multiple trauma*" OR "major trauma*" OR "multiple injur*" OR "multisystem trauma" OR "multisystem injur*" OR multitrauma OR "trauma patient*" OR "trauma population" OR "trauma care" OR "trauma cent*" OR "critical illness*" OR "critically ill*" OR "critical care" OR "intensive care" OR (blunt AND trauma))) OR AB=((polytrauma* OR "multiple trauma*" OR "major trauma*" OR "multiple injur*" OR "multisystem trauma" OR "multisystem injur*" OR multitrauma OR "trauma patient*" OR "trauma population" OR "trauma care" OR "trauma cent*" OR "critical illness*" OR "critically ill*" OR "critical care" OR "intensive care" OR (blunt AND trauma)))

#4

#1 AND #2 AND #3

#5.

TI=(random* OR placebo OR trial OR groups) OR AB=(random* OR placebo OR trial OR groups)

#6

TI=(meta analysis OR systematic review) OR AB=(search* OR meta analysis OR medline OR systematic review)

#7

#5 OR #6

#8

#4 AND #7  
= 388
